# Pathogenesis and virulence of herpes simplex virus

**DOI:** 10.1080/21505594.2021.1982373

**Published:** 2021-10-22

**Authors:** Shuyong Zhu, Abel Viejo-Borbolla

**Affiliations:** Institute of Virology, Hannover Medical School, Cluster of Excellence RESIST (Exc 2155), Hannover Medical School, Hannover, Germany

**Keywords:** Herpes simplex virus, pathogenesis, virulence, herpes stromal keratitis, genital herpes, herpes simplex encephalitis, herpes and Alzheimer’s disease

## Abstract

Two of the most prevalent human viruses worldwide, herpes simplex virus type 1 and type 2 (HSV-1 and HSV-2, respectively), cause a variety of diseases, including cold sores, genital herpes, herpes stromal keratitis, meningitis and encephalitis. The intrinsic, innate and adaptive immune responses are key to control HSV, and the virus has developed mechanisms to evade them. The immune response can also contribute to pathogenesis, as observed in stromal keratitis and encephalitis. The fact that certain individuals are more prone than others to suffer severe disease upon HSV infection can be partially explained by the existence of genetic polymorphisms in humans. Like all herpesviruses, HSV has two replication cycles: lytic and latent. During lytic replication HSV produces infectious viral particles to infect other cells and organisms, while during latency there is limited gene expression and lack of infectious virus particles. HSV establishes latency in neurons and can cause disease both during primary infection and upon reactivation. The mechanisms leading to latency and reactivation and which are the viral and host factors controlling these processes are not completely understood. Here we review the HSV life cycle, the interaction of HSV with the immune system and three of the best-studied pathologies: Herpes stromal keratitis, herpes simplex encephalitis and genital herpes. We also discuss the potential association between HSV-1 infection and Alzheimer’s disease.

## Introduction

Herpes simplex virus type 1 (HSV-1), and HSV-2 are highly prevalent human pathogens with worldwide prevalence levels of about 67% and 13%, respectively [1]. It is estimated that in 2016 approximately 3.7 billion people worldwide were seropositive for HSV-1 and nearly 500 million for HSV-2 [1]. Transmission of both HSV-1 and HSV-2 occurs through close contact and results in a lifelong infection. Most people acquire HSV-1 early in life through the orolabial mucosa, while HSV-2 infections occur later, normally through sexual transmission. Infection with one HSV type normally induces immunity to prevent re-infections with the same serotype, but not with the other [2].

The outcome of infection with HSV-1 and HSV-2 can be asymptomatic, mild or life-threatening. In most immunocompetent individuals HSV causes mild and self-resolving disease. However, HSV infection is also associated with high morbidity and mortality in certain individuals for reasons that are not completely understood. Diseases caused by HSV include cold sores, genital herpes, herpes stromal keratitis (HSK), eczema herpeticum, disseminated disease in the neonate, meningitis and herpes simplex encephalitis (HSE). Several reports suggest also a link between HSV infection and neurodegenerative diseases. The interaction between HSV and the host, in particular with the immune system, determines the outcome of infection. Genetic defects in intrinsic and innate defense mechanisms in the CNS are linked to higher risk of suffering HSE [3]. Individuals with deficient T cell immunity are more prone to recurrent meningitis, pneumonitis and hepatitis [4]. Neonatal infection is more aggressive than that of adults, partly due to the lack of a mature immune system, and results in systemic viral dissemination with high mortality and morbidity rates if untreated [5–7]. Finally, there seems to be an association between HSV-1 infection and Alzheimer’s disease (AD) mainly in people that harbor the apolipoprotein E ε4 (APOE4) allele. These observations clearly show that both the innate and adaptive immune responses are fundamental to control HSV infection and reduce pathogenesis. They also show that host genetic polymorphisms account for some of the most severe forms of disease. On the other hand, HSV is very well equipped with virulence factors that modulate and evade the immune response [8,9]. Moreover, an excessive and uncontrolled response of the immune system can contribute to pathogenesis as observed in HSK [10,11].

## HSV cell cycle

**Figure 1.**
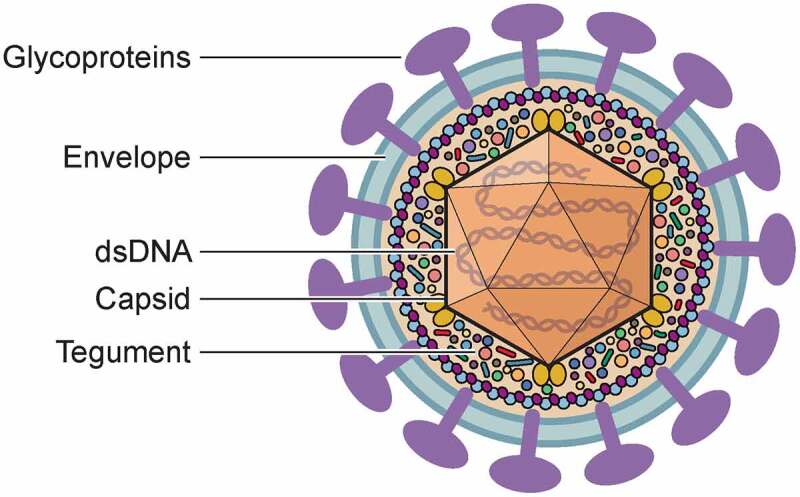
**The HSV virion**. The linear double stranded DNA forms the core of the virion and is protected by the icosahedral capsid. The tegument, composed of many viral and cellular proteins surrounds the capsid and connects it with the envelope, where the viral glycoproteins and other membrane associated proteins are embedded

HSV-1 and HSV-2 contain a large, linear double stranded DNA genome protected by an icosahedral capsid surrounded by a proteinaceous layer termed the tegument and wrapped in an envelope containing viral glycoproteins (Figure 1). Initial attachment to the plasma membrane occurs through binding of glycoprotein B (gB) and gC to glycosaminoglycans (GAG) [12]. HSV gG also binds to GAGs but its role in virus attachment has not been established [13,14]. Binding to GAGs is followed by interaction of gD with several entry receptors: herpesvirus entry mediator (HVEM), nectin-1 and −2 and 3-O-sulfated HS [15]. Several reports showed that the interactions between gB and paired immunoglobulin-like type 2 receptor α (PILRA), myelin-associated glycoprotein and non-muscle myosin IIA are also involved in HSV entry [16–18]. The interaction of HSV-1 gH/gL with specific integrins leads to HSV-1 entry through endocytosis [19]. The expression of the receptors differs between tissues and cell types, influencing virus tropism. For instance, HVEM and nectin-1 seem to be the main receptors in the cornea and the nervous system, respectively [20–22]. Interestingly, HSV-1 requires HVEM to infect the mouse cornea, while HSV-2 does not [23]. Mice lacking HVEM and nectin-1 are resistant to HSV-1 and HSV-2 induced pathogenesis showing the relevance of these receptors for HSV infection [20,21].

**Figure 2.**
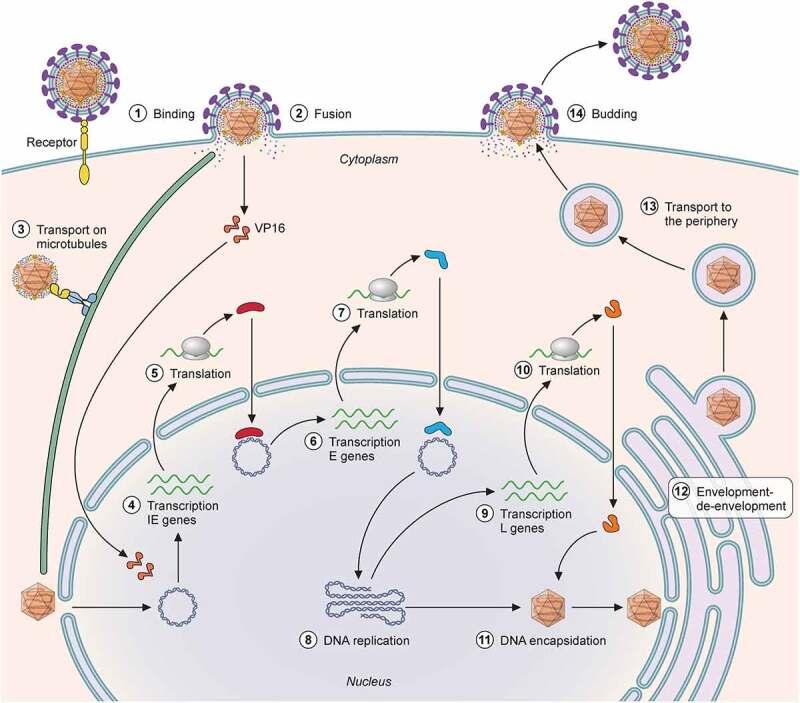
**HSV cell cycle**. (1) HSV glycoprotein D or B interact with specific cellular receptors leading to fusion at the plasma membrane (2) of following endocytosis (not shown in this figure). Upon fusion, the capsid is released to the cytoplasm with some attached tegument proteins, while other tegument proteins like VP16 separate from the capsid. (3) The capsid travels to the cell nucleus using microtubuli due to the interaction between UL36 and motor proteins. The linear DNA enters the nucleus . (4) The tegument protein VP16 enters the nucleus together with HCF-1 and Oct-1 and starts transcription of IE genes. (5) The IE genes are translated and participate in the transcription of E genes (6), which take part in the replication of the viral genome (8). Once there are sufficient copies of viral genomes, the products of the L genes facilitate DNA encapsidation (11). The mature, DNA containing capsids (C capsids) leave the nucleus through an envelopment-deenvelopment process and acquire tegument and envelope (not shown) prior to cellular egress (14)

Interaction with the cellular receptor(s) triggers binding of gD to a gH/gL heterodimer and exposure of the gB fusion peptide, leading to fusion of the viral and cellular membranes [24]. Fusion can take place at the plasma membrane or within vesicles following viral internalization. Following fusion, some tegument proteins, like VP16, dissociate from the capsid and travel to the nucleus independently [25], while others remain bound (Figure 2). Inner tegument proteins mediate interaction with dynein, dynactin and kinesin motor proteins and facilitate capsid transport on microtubules toward the nucleus [26–30]. Most evidence points to pUL36 and pUL37 as the main viral proteins involved in nuclear targeting required for import of the genome into the nucleus [27,29,31].

The viral linear DNA genome enters the nucleus through a nuclear pore [32] (Figure 2). The cellular RNA polymerase II and viral proteins transcribe HSV genes. Gene expression follows an ordered cascade during lytic replication. Immediate early (IE) genes – infected cell protein (ICP) 0, ICP4, ICP22, ICP27, ICP47 and unique short (US) 1.5 – are expressed in the absence of *de novo* viral protein synthesis. The tegument protein VP16 forms a complex with host cell factor 1 (HCF-1) and octamer binding protein-1 (Oct-1) that binds to the promoter of IE genes, driving their expression [33]. One of the roles of IE genes is to drive the transcription of early (E) genes, whose many of their products are proteins involved in DNA replication that ensues through the rolling circle mechanism. Once DNA replication occurs, late (L) genes are expressed. Many of the L genes are structural proteins involved in virus assembly.

Viral transcription, DNA replication, capsid assembly and DNA encapsidation occur exclusively in the nucleus [34]. Cellular proteins, such as importin alpha, are required for efficient nuclear import of viral proteins and for capsid assembly and egress [35]. Mature capsids containing viral DNA leave the nucleus through an envelopment-deenvelopment process (reviewed in [36]). Briefly, the capsid obtains a primary envelope from the inner nuclear membrane. This envelope is lost upon fusion with the outer nuclear membrane and release of the capsid into the cytoplasm. The nuclear egress complex formed by pUL31 and pUL34 mediates this process through interaction with viral and cellular proteins like lamin A/C [37–41]. Following exit from the nucleus, cytosolic capsids acquire more inner tegument proteins, while outer tegument proteins and viral membrane proteins are incorporated at the membrane compartments of trans-Golgi network vesicles and endosomes [42,43]. There is discussion on whether certain tegument proteins are incorporated into the capsid inside the nucleus [27,44–49]. Inner tegument proteins, mainly pUL36, pUL37 and pUS3 associate with the capsid first, and outer tegument proteins do so later [44,50,51]. pUL36 and pUL37 direct the movement of capsids on microtubules toward the site of secondary envelopment [52–57]. Other HSV proteins such as pUL20 and gK also participate in secondary envelopment [58,59]. pUL36 and pUL37 are required for HSV transport from the nucleus toward the periphery through interaction with kinesin [27,52,54,60,61]. The mechanisms leading to transport and incorporation of viral glycoproteins are not completely understood. This process is particularly important and interesting in highly polarized cells like neurons. It is not clear whether fully enveloped capsids form inside the cell body or whether naked capsids and envelope proteins are transported independently and envelopment occurs in the axons (reviewed in [62–64]). Recent data showed that pUL36 and pUL37 mediate motility in the neuronal cell body but cannot direct the non-enveloped capsids to the axons, contrary to vesicles containing gD that efficiently employed axonal transport [61]. These results suggest that only fully assembled viral particles can travel from the cell body to the axon termini. Vesicles transport HSV particles to the plasma membrane and enveloped HSV exits the cell upon fusion of the vesicle with the plasma membrane. For a recent review on HSV egress see [49].

## Primary infection – from epithelial cells to neurons

**Figure 3.**
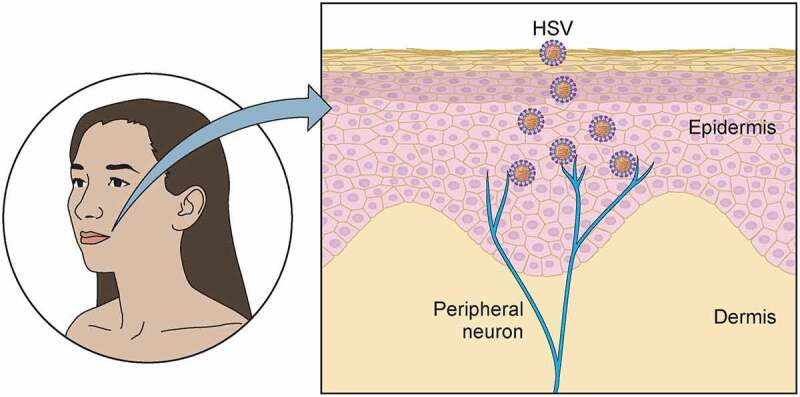
**Initial steps of HSV primary infection**. During primary infection, HSV infects epithelial cells in the mucosa or skin. Infection of the skin requires rupture of the keratin layer composed of dead cells. The virus replicates lytically in epithelial cells producing new infectious viral particles that reach nerve endings of peripheral neurons, where HSV establishes latency

During primary infection, HSV infects epithelial cells in the mucosa or skin and then establishes latency in neurons, mainly of the peripheral nervous system (PNS, Figure 3). Infection of the central nervous system (CNS) could lead to acute infection and inflammation associated with high morbidity and mortality. However, HSV DNA has been found in human CNS and HSV-1 can establish latency in human brain organoids [65,66], suggesting that a similar process could occur *in vivo*.

Transmission of HSV-1 and HSV-2 between individuals occurs through close contact. Infection through the skin normally requires prior damage to the apical layers of this protective organ. For instance, HSV gains access to epithelial cells in the mucosa or epidermis thanks to microscopic ruptures that occur during coitus or by abrasion of the skin. Cell-to-cell virus spread is very important for transmission in the skin and a study suggested that HSV induced polarization of the non-infected cells toward the infected ones by an unknown mechanism [67]. Infection of the skin or the mucosa is accompanied by inflammation and tissue damage, causing the characteristic herpes blisters. HSV does not spread systemically in immunocompetent individuals. Following efficient replication in epithelial cells, HSV reaches nerve endings of peripheral neurons and undergoes retrograde transport to the neuronal cell body (Figure 3). Free nerve endings are dynamic axonal termini that can retract or degenerate when exposed to attractive or repulsive, respectively, axon guidance molecules, neurotrophic factors and cytokines [68]. Such cues are expressed during infection and inflammation. Many pro-inflammatory cytokines like interleukin (IL)-6 and tumor necrosis factor (TNF)-α repel neurites. Members of the semaphorin, netrins, ephrin and slit families repel or attract neurites, depending on their intrinsic properties and the cellular context [69]. The neurotrophic factors like nerve growth factor (NGF) and the cytokine IL-17C induce neurite outgrowth. HSV-2 has developed mechanisms to facilitate neurite outgrowth. For instance, HSV-2 gG binds and enhances the activity of NGF, increasing neurite outgrowth [70,71]. Moreover, infection of non-neuronal cells by HSV-2 reduces their repelling effect on neurite outgrowth [71]. Whether this impacts colonization of neurons responsive to NGF is currently unknown. Interestingly, HSV-2 also induces IL-17C expression in keratinocytes of the human genital tract during reactivation, leading to neurite outgrowth [72]. The authors suggested that the higher level of IL-17C increased neuronal survival during recurrent HSV-2 reactivation [72]. Other HSV proteins including gD, gK, gE, pUL36 and pUL37 are required for efficient virus colonization of neurons from the periphery [31,73–80]. Moreover, other proteins like ICP34.5 and US11 are neurovirulence factors [81–83].

## Latency and reactivation

HSV infection results in either lytic or latent replication. During lytic replication there is an orchestrated expression of viral genes leading to production of infectious virus, while during latency there is limited gene expression and no production of viral particles. However, the viral genome is competent for reactivation, leading to the production of infectious virions upon the appropriate stimulus. HSV latency and reactivation have been studied employing different models, all with advantages and disadvantages (reviewed in [84]). HSV establishes latency in neurons. Infection of susceptible non-neuronal cells normally leads to lytic replication, although a recent report suggested the existence of latency in a proportion of non-neuronal cells *in vitro* [85]. Whether a similar phenomenon occurs *in vivo* requires further investigation.

**Figure 4.**
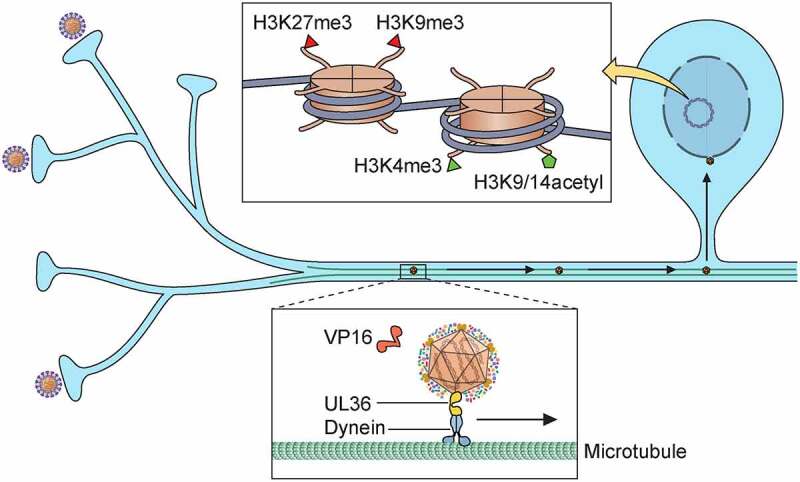
**Establishment of HSV latency in neurons**. Following entry in the neurite end the capsid containing pUL36 and other inner tegument proteins travel to the nucleus independently of other tegument proteins like VP16. The transport of VP16 is not efficient and it probably reaches the nucleus later than the viral DNA. This, together with other factors, leads to the deposition of histone H3 and subsequently the addition of constitutive and facultative heterochromatic marks (H3K9me3 and H3K27me3, respectively) on most viral promoters, repressing their transcription. On the contrary, the LAT locus contains facultative heterochromatin and euchromatin marks (H3K4me3 and H3K9/14acetyl), facilitating its transcription

The reasons why HSV-1 and HSV-2 establish and maintain latency in neurons but not in other cell types are not completely clear. Experiments performed with murine neurons grown in microfluidic chambers showed that infection at the neuronal cell body resulted in production of infectious viral particles, while infection at the axons led to nonproductive infection, especially if the number of infectious viral particles was low [86–88]. Interestingly, axonal infection was productive if complemented at the cell body area with helper virus containing VP16, showing the relevance of this tegument protein in the initiation of the lytic transcripts and restricting silencing of viral gene expression [86]. Following fusion of the HSV envelope and a cellular membrane, some tegument proteins, including VP16, travel to the nucleus independently from the capsid and do not reach the neuronal nucleus when the genome does [25,89], probably due to the long distance from the neurite end to the cell body (Figure 4). The low levels of VP16, of other tegument proteins like ICP0 and of newly expressed genes required to inhibit the repressive activity of cellular proteins, might lead to inefficient viral gene expression and entry into latency in neurons [90–92]. These results suggest that an efficient expression of IE genes is required to avoid establishment of latency. However, in mouse TG neurons, there is initial lytic gene expression and replication during the acute phase of infection prior to establishment of latency [93,94]. VP16 seems to be required for the initial lytic gene expression and for transition from latency into lytic replication, probably due to the presence of Egr-1/Sp1 binding sites in the VP16 promoter that facilitate its expression in neurons early upon infection [95,96]. Interestingly the VP16 promoter of HSV-2 contains more Egr-1/Sp1 binding sites than that of HSV-1. Introduction of the HSV-2 VP16 promoter sequence in the HSV-1 background led to a more virulent virus that reactivated more efficiently and caused higher mortality in mice upon corneal infection [96]. HSV-1 mutants without VP16 transactivating properties could not reactivate efficiently in mice [97,98], supporting a role for this viral protein in the transition between latent and lytic cycles. However, VP16 expression did neither block entry into latency nor induced reactivation in mouse sensory neurons *in vivo* [99,100]. Therefore, more research is required to solve the role of VP16 during latency establishment and reactivation. ICP0 might also participate in the transition from latency to lytic replication since it affects cellular activities that result in modifications of histone marks and viral gene expression, possibly contributing to reactivation [101,102].

Data from animal models and *in vitro* suggest that the view of latent and lytic cycles as mutually exclusive is perhaps not completely correct, and that there are intermediate stages with different levels of viral gene expression [103]. There is heterogeneity in the ganglion with HSV undergoing lytic transcription that may be blocked by the host, or lead to production of infectious virions in some neurons while being latent in others [103]. Moreover, some genes considered lytic might be expressed as part of a latency program [104]. For instance, genetic studies in mice indicated that the IE gene ICP0 is expressed during latency and contributes to the structure of latent viral chromatin [105]. The different outcomes of infection probably depend on the interactions between the infected neurons, satellite glial cells and cells of the innate and adaptive immune response, including macrophages and T cells [106]. The use of single-cell approaches to study latency and reactivation will provide relevant information in these interesting processes. One such study with reporter mice and HSV-1 expressing *Cre* recombinase to analyze individual neurons showed that expression of IE and E genes prior to establishment of latency did not result in increased copies of viral DNA [94]. However, there were more reactivation events in cells containing higher viral loads [94]. Another study found lytic viral gene expression and transcripts of cellular genes involved in intrinsic immunity in latently infected mouse neurons, suggesting attempts of HSV to reactivate and potential control by the host [107]. The fact that HSV-specific T cells are found in animal and human sensory ganglia [108–111] suggests that translation of some transcripts could occur during establishment and maintenance of latency or in reactivation episodes. However, the viral transcripts found by RNA-seq in infected human TG obtained at a short postmortem interval are restricted to the latency-associated transcripts (LATs) and microRNAs expressed from the LAT locus [112]. The literature on LATs and its role in the establishment of latency and reactivation is very extensive and reviewed elsewhere [113,114]. Briefly, expression of LATs seems to promote stable but reversible silencing of the HSV genome [115], and the cellular microRNA 138 and viral microRNAs inhibit translation of HSV transcripts, probably supporting latency [116,117]. The different levels of LAT expression in different neurons might also influence establishment and maintenance of latency.

Epigenetic modifications regulate establishment and maintenance of latency and reactivation at the molecular level [118]. The incoming HSV genome lacks histones and methylation. Once the viral DNA enters the cell and is sensed by pattern recognition receptors (PRR, see “Intrinsic and innate immune responses against HSV”), several enzymes deposit histones and histone modifications on the HSV genome, restricting gene expression. Interestingly, methylation of the HSV genome does not seem to be involved in latency and reactivation [119]. During the lytic cycle, histones 3 (H3) containing repressive marks decorate the incoming HSV-1 genomes from one hour post-infection [120,121] and inhibit the transition from IE to E gene expression [122]. However, some viral proteins decrease heterochromatin levels and increase euchromatin marks to facilitate viral gene expression. On the contrary, the latent genome contains constitutive and facultative heterochromatin marks on most viral genes while the LAT locus has facultative heterochromatin and euchromatin modifications [123–126]. Chromatin insulators mediate the separation of transcriptionally active and repressed regions and facilitate reactivation [127,128].

Interferon (IFN)-γ-inducible protein 16 (IFI16) and promyelocytic leukemia (PML) nuclear bodies (NB) are two elements of the intrinsic immune response involved in the epigenetic regulation of HSV gene expression. Both bind to HSV DNA and promote its heterochromatinization, silencing the viral genome [129–131]. PML-NB colocalize with incoming HSV-1 genomes, contributing to their chromatinization, and this association remains during latency in mouse sensory neurons [126,132,133]. PML-NBs contain more than 70 cellular proteins involved in many activities, including chromatin modification and regulation of gene expression, and in antiviral response [134]. IFI16 forms filamentous structures on HSV DNA that recruit components of PML-NB including PML, speckled 100 kDa (SP100) and alpha thalassemia/mental retardation syndrome X-linked (ATRX), to increase repression of viral gene expression [129,135–137]. PML-NB proteins, like death domain associated protein (DAXX) and ATRX, are essential for H3.3 deposition at heterochromatin loci, including HSV-1 promoters [126,138,139]. The histone cell regulator (HIRA) is a histone H3.3 chaperone that deposits H3.3 onto the HSV-1 genome while ATRX aids to maintain the heterochromatin marks on the histones [140,141]. On the contrary, during lytic replication HIRA participates in the addition of H3.3 with euchromatin marks on HSV-1 genes, facilitating viral gene expression [142]. The RE1-silencing transcription factor (REST)/CoREST/histone deacetylases (HDAC) nuclear repressor complex contributes to silence the HSV-1 genome and to regulate latency and reactivation [122].

As mentioned above, the reduced level of VP16, ICP0 and other viral proteins probably contributes to entry into latency. Many of these viral proteins counteract the activity of the restrictive epigenetic regulators and increase the deposition of euchromatic marks on the viral genome, leading to viral gene expression. For instance, ICP0 dissociates histone deacetylases from the REST/CoREST complex, facilitating viral gene expression [122,143]. The VP16/HCF-1/Oct-1 complex also binds to REST/CoREST/HDAC nuclear repressor complex, leading to the removal of heterochromatin and facilitating the addition of euchromatin marks on histones associated with IE genes. Since IFI16 and PML-NBs are also heavily involved in the intrinsic immune response and the induction of innate immune responses dependent on IFN (see “Intrinsic and innate immune responses against HSV”), the inhibition of their activities by ICP0, VP16 and other viral proteins is also relevant as an immune evasion strategy.

The viral proteins involved in driving viral gene expression during lytic replication, such as VP16, are not expressed during latency. Moreover, the latent chromatin structure contains repressive heterochromatin marks and is associated with nucleosomes [144]. Therefore, this raises the question of how viral gene expression starts during reactivation from a silenced genome. The answer to this question could be in the connection between the neuronal stress response and the c-Jun N-terminal kinase (JNK) pathway. Moreover, the VP16 promoter contains Egr-1/Sp1-binding sites that could respond to transcription factors expressed during neuronal stress response [95]. This could facilitate VP16 expression and thereby reactivation in neurons during stress conditions. Several stimuli that cause neuronal stress induce reactivation in animal models and in cell culture. These include removal or inhibition of neurotrophic factors, exposure to UV light, inhibition of phosphoinositide 3-kinase (PI3K), inhibition of histone deacetylases and activation of JNK. In humans, exposure to UV light, changes in hormone levels and fever can trigger HSV reactivation [145]. One cytokine expressed in these circumstances, IL-1β, has been shown to induce HSV-1 reactivation from latently infected mouse neurons in a dual-leucine-zipper kinase (DLK)-dependent manner [146]. Neuronal stress response leads to activation of the JNK pathway by JNK interacting protein 3 (JIP3) and DLK, inducing HSV-1 gene expression [147]. Whether IL-1β plays a similar role in humans is currently unknown. The mechanisms by which JNK induces gene expression from a repressed genome seem to involve the phosphorylation of serine residues in histones, allowing gene expression even in the presence of repressive methylation marks on histone lysine residues, in a process termed methyl-phospho switch [147].

The work with murine neurons suggests that reactivation can be divided in two phases. In the first one, termed animation, there is low level of genome-wide expression in the absence of viral proteins, leading to protein translation [104,148]. This viral gene expression upon reactivation *in vitro* or upon explanting infected neurons from mice and removal of NGF does not seem to follow the ordered cascade of IE, E and L genes observed during acute infection [148,149]. VP16 expression is required for the second phase of reactivation, inducing the expression of IE genes and starting a similar expression cascade as during lytic infection [148].

Following reactivation, new infectious viral particles are produced. These particles travel in an anterograde manner to the skin or mucosa causing the typical herpes lesions. The virus may also travel to the CNS where it could cause encephalitis or meningitis. HSV reactivation is more frequent than initially anticipated, as shown by studies detecting virus in the oral and genital mucosa of asymptomatic individuals [150–153].

## Intrinsic and innate immune responses against HSV

The infection of the first few cells of the host triggers many intra- and intercellular responses that must be conveyed and coordinated to control HSV infection. The intrinsic and innate immune responses are critical during these initial stages. The intrinsic immune response is composed of preexisting, constitutively active restriction factors that inhibit infection immediately, without requiring the expression of IFN and IFN stimulated genes (ISG). An important role of the intrinsic immune response against HSV, already discussed above, results in repression of HSV gene expression. The innate immune response requires the action of cytokines, including IFN, and ISG. Both responses are interconnected and are sometimes difficult to separate. For instance, the expression of several proteins involved in the intrinsic response can be enhanced by IFN. Moreover, the intrinsic immune response can lead to the expression of IFN and other cytokines. Therefore, we will discuss both types of responses together in this review. The intrinsic and innate immune responses are highly effective against HSV during primary infection and to control reactivation from latency. They also contribute to an efficient adaptive immune response to HSV. However, despite this, reactivation occurs and is quite frequent in certain individuals, probably due to a combination of viral evasion mechanisms and lack of effective immune control.

To detect pathogens, the cells express PRRs that sense pathogen-associated molecular patterns (PAMPs) and damage associated molecular patterns and signal through adaptor proteins such as TIR-domain-containing adapter-inducing interferon-β (TRIF) and mitochondrial antiviral-signaling protein (MAVS) to initiate the innate immune response [154,155]. This normally leads to the activation of signaling cascades including IFN regulatory factor (IRF) family members that result in IFN expression. Other activated pathways are the nuclear factor kappa light chain enhancer of activated B cells (NFκB) and activating protein 1 that in most occasions induce the expression of cytokines other than IFN. These signaling cascades might converge and show certain redundancy. Among the cytokines expressed are members of the IL, TNF, chemokine and IFN families. These cytokines can act in autocrine and paracrine manners and are essential to control HSV during primary infection and upon reactivation and to coordinate the innate and adaptive immune responses. They orchestrate the recruitment of immune cells, the maturation of the adaptive immune response and participate in its resolution. When uncontrolled, the inflammatory response can have detrimental effects, contributing to pathogenesis.

Most of the signaling cascades activated by PRRs upon recognition of HSV lead to the expression of IFNs, one of the main cytokine families inhibiting HSV. There are three types of IFNs described to date: the multi-gene cytokine family of type I IFN containing IFN-α and β as prototypes, type II IFN including only IFN-γ and type III IFN containing IFN-λ1-4 [156–158]. Most types of human cells express type I IFNs, while IFN-γ is expressed mainly by T and NK cells. Type III IFNs are expressed by many cell types, although myeloid cells seem to be the main producers. Each type of IFN binds to a different IFN receptor and activates signaling cascades that, despite certain differences, involve the Janus kinase signal transducer and activator of transcription (JAK/STAT) pathways. IFN-α and IFN-λ receptors signal through the transcription factor complex IFN-stimulated gene factor 3, whereas IFN-γ receptor normally signals through gamma activated factor. The outcome of IFN signaling is the expression of IFN-stimulated genes (ISGs), which are not identical for each IFN and cell type, generating an antiviral response [159].

PRRs sense HSV at different stages of its lifecycle: upon cell binding, during viral fusion, capsid transport, genome release and after the generation of RNA intermediates. Toll-like receptors (TLRs) are PRRs that detect PAMPs in nucleic acids and proteins. There are 12 TLRs identified in mammals and TLR2, 3 and 9 are the main ones sensing HSV [155,160]. TLR2 detects viral glycoproteins during HSV binding or fusion, TLR3 senses dsRNA produced as byproducts of HSV replication and TLR9 recognizes HSV DNA. Other cytoplasmic and nuclear sensors also detect HSV DNA and RNA intermediates. Sensing of HSV nucleic acids is key to control the virus. Individuals with errors in DNA sensors, the RNA sensor TLR3, or downstream signaling pathways tend to suffer severe HSE (reviewed in [161,162]). The relevance of TLR3 during HSV infection is discussed in more detail below (see “Herpes simplex encephalitis”).

TLR2 detects viral gH and gL present on the viral envelope upon HSV interaction with the plasma membrane and signals through myeloid differentiation factor 88 (MyD88) [163,164]. This leads to the activation of TRIF-related adaptor molecule and type I IFN expression and/or to the activation of TNF receptor associated factor (TRAF), NFκB and expression of pro-inflammatory cytokines [163,165,166]. The expression of pro-inflammatory cytokines upon TLR2 activation induces an effective antiviral response [167] but might be detrimental for the host, since TLR2-deficient mice have less leukocyte infiltration, less symptoms and survive more than wild type mice, despite efficient HSV-1 replication in the brain [165,168]. The fusion of the HSV envelope with a cellular membrane is also sensed by PRR, triggering calcium signaling, activation of the stimulator of IFN genes (STING), IRF3 and IFN response [169].

TLR9 detects HSV DNA in endosomes. The relevance of TLR9 has been shown *in vitro*, and seems to be particularly important is certain cell types, like plasmacytoid dendritic cells (pDCs), where TLR9 ablation results in a deficient IFN response [170,171]. However, TLR9 is not essential against HSV in murine models of infection [166,172]. The viral capsid protects the DNA during cytoplasmic transport to the nucleus. Despite this, several cytoplasmic sensors detect HSV DNA. One mechanism observed in macrophages involves the degradation of HSV capsids, releasing HSV DNA to the cytoplasm, facilitating its detection [173]. Cyclic guanosine monophosphate-adenosine monophosphate synthase (cGAS) and IFI16 detect HSV DNA and activate STING, leading to recruitment of TANK-Binding Kinase 1 (TBK1), activation of IRF3 and induction of IFN expression [136,174–177]. cGAS binds to dsDNA in a sequence-independent manner, leading to the synthesis of the second messenger cyclic G(2ʹ−5ʹ)pA(3ʹ−5ʹ)p (2ʹ3’-cGAMP) that binds STING [178,179]. IFI16 mainly detects and binds HSV DNA in the nucleus, leading also to IFN production. IFI16 also induces IFN expression through interaction with its promoter [136]. Moreover, IFI16 binding to HSV DNA restricts its gene expression as explained above (see “Latency and reactivation”) and contributes to the inflammasome response, inducing pro-caspase-1 activation [130]. Genetic evidence in humans also supports a role for IFI16 in protecting against genital herpes caused by HSV-2 [174]. The cGAS-STING pathway is essential to control HSV-1 brain infection as shown with mouse models [179,180] and human genetic studies (see “Herpes simplex encephalitis”). STING is expressed in most cells, in contrast to TLRs. Activation of STING with agonists induces type I IFN and reduces HSV-2 replication and disease, without triggering a detrimental inflammatory response in human cells and in mouse models [181]. Another DNA sensor, absent in melanoma 2 (AIM2) also detects HSV and activates the inflammasome, resulting in caspase-1 cleavage of cytokine precursors IL-1β and IL-18 [182]. The role of AIM2 in protection against HSV is not well understood (reviewed in [183]). Genetic polymorphisms in the DNA sensor RNA polymerase III increase susceptibility to varicella zoster virus (VZV) in humans without affecting HSV replication or immune response [184].

As indicated above, intrinsic and innate immune responses are interconnected. The interaction of the HSV genome with PML-NB occurs early upon infection and facilitates restriction of viral expression prior to the launch of an IFN response [133]. Several proteins within PML-NB act as HSV restriction factors and their depletion results in higher viral gene expression and replication of a virus lacking ICP0, showing the relevance of this tegument protein to inhibit PML-NB activity [185–188]. ICP0 is an E3 ligase [189] that targets several proteins for degradation, including IFI16, ATRX, Sp100 and PML [175,187,190], clearly indicating the relevance of these proteins in the anti-HSV response. Deletion of ICP0 renders HSV susceptible to PML-NB, and this effect is increased by IFN [185,191]. The interaction of PML-NB with other cellular proteins also contributes to the induction of IFN and ISG. Sensing of HSV leads to the expression of cytokines resulting in HIRA interaction with PML-NBs in a JAK, cyclin-dependent kinase and Sp100-dependent manner. This increases the innate immune response against HSV-1 by inducing the expression of antiviral genes, including cytokines and ISG [192].

Several PRR detect HSV RNAs produced as intermediate products of HSV replication. Endosomal TLR3, cytosolic RNA helicases and retinoic acid-inducible gene I (RIG-I) like receptors (RLR) detect HSV dsRNAs [172,193]. Recognition of dsRNA by melanoma differentiation-associated protein 5, a member of the RLR family, is followed by activation of MAVS and induction of IFN and other cytokines in HSV-infected primary human macrophages [193]. IFN induces protein kinase RNA-activated (PKR) that detects HSV dsRNA in the cytoplasm. This results in the phosphorylation of eukaryotic initiation factor 2 alpha, blocking translation and triggering autophagy [194,195]. PKR binding to dsRNA also induces the production of IFN and other cytokines through the NFkB pathway [196]. Both PKR and IFN can induce autophagy, a cellular response to starvation that results in the degradation of proteins and organelles by autophagosomes and fusion with lysosomes. Autophagy acts as an important immune response in limiting viral replication, especially in neurons [82,197,198].

HSV employs several strategies to counteract the antiviral activity of the intrinsic and innate immune responses. Some of the viral proteins involved in these immunomodulatory activities are indicated below. For more detailed reviews on HSV modulation of the innate immune response see [183,199]. Many of these HSV proteins are virulence factors, increasing pathogenicity. HSV-1 pUS3 inhibits TLR2 signaling pathway by reducing TRAF6 ubiquitination and thereby the NFκB pathway [200]. It also inhibits the activation of type I IFN by TLR3, as shown with human monocytes [201]. The tegument protein VP22 interacts with cGAS, inhibiting the formation of cGAMP and thereby type I IFN production [202]. pUL37 deaminates and inactivates cGAS [203] and the helicase domain of RIG-I, impairing its ability to recognize dsRNA and to inhibit HSV replication [204]. The deubiquitinase (DUB) activity of pUL36 is required to reduce type I IFN production in mice [205]. HSV-1 lacking DUB activity replicates less efficiently in the mouse brain and induces higher level of IFN in human microglia [205]. Moreover, lack of DUB increases STING ubiquitination and subsequent phosphorylation of STING, TBK1 and IRF3 [205]. Deubiquitination of STING leads to reduced phosphorylation of this protein, TBK1 and IRF3 and thereby lower expression of IFN-β [205]. pUL36 also deubiquitinates TRAF3, required for the induction of IFN through TLR3 [206]. ICP27 interacts with TBK1-STING in human macrophages, impairing IRF3 phosphorylation and downstream activation of STING, MAVS and TRIF [207]. ICP34.5 inhibits STING activation [208]. Overexpression of *UL46* reduces STING expression and IFN production, probably by reducing TBK1 dimerization and subsequent interaction with IRF3 [209,210]. The product of UL49, VP22, blocks IFI16-mediated pro-caspase activation inhibiting the secretion of pro-inflammatory cytokines [211]. HSV-1 virus host shutoff protein (VHS) reduces cGAS and IFI16 mRNA levels and ICP0 targets these proteins for degradation by the proteasome, reducing IFN production [130,212,213].

A mechanism employed by HSV to inhibit NFκB signaling is to block the nuclear translocation of the p50/p65 complex to the nucleus. It does so by impeding the degradation of IκBα and through phosphorylation of p65 that reduces its translocation to the nucleus [214]. The inner tegument protein pUL36 deubiquitinates IκBα and thereby inhibits its degradation [215]. HSV US3 blocks the nuclear translocation of NFκB induced by TLR2 by blocking the ubiquitination of TRAF6 [200]. ICP0 polyubiquitinates and targets p50 for degradation, while ICP27 inhibits the phosphorylation and ubiquitination of IκBα [216,217]. pUL24 also blocks p50/p65 translocation through binding to Rel homology domains [218]. VP16 binds NFκB subunits and impedes the activation of the NFκB promoter [219].

VHS, US11 and ICP34.5 inhibit PKR activity by degrading mRNA, competing for dsRNA binding and by dephosphorylating eIF2a [220–222]. PKR and IFN signaling can induce autophagy and ICP34.5 inhibits this cellular process, contributing to neuropathology, including encephalitis in mice. ICP34.5 blocks PKR-induced autophagy through interaction with Beclin 1 [82].

Several proteins inhibit the activation of IRF3. For instance, VP24 blocks the interaction between TBK1 and IRF3 [223], while ICP34.5 impairs IRF3 phosphorylation [224,225]. VP16 inhibits IRF3 activation of IFN response by impeding the recruitment of the CREB-binding protein coactivator [219]. HSV-1 US3 binds to and phosphorylates IRF3 preventing its activation and blocking type I IFN production [226]. ICP0 blocks the activation of IRF3 and IRF7, reducing the expression of ISG [227,228]. Similarly, several proteins block IFN and ISG activity. US3 and pUL13 block the activation of the IFNGR1, by reducing its phosphorylation [229]. Moreover, pUL36 inhibits the interaction between IFNAR and JAK1, blocking subsequent signaling [230]. VHS reduces JAK and STAT2 levels and transcripts of several ISGs [231–233].

## Adaptive immune response to HSV

The adaptive immune response is very important to control HSV infection and reactivation. The role of T cells is particularly relevant since HSV-specific T cells are present in infected sensory human ganglia and in active and resolved lesions of patients [110,111,234–236]. The percentage of HSV-specific T cells in blood of immunocompetent individuals after resolution of acute infection is low [237,238]. Blood CD8 T cells specific for HSV express high levels of cytolytic molecules and secrete IFN-γ when exposed to viral antigen [239]. Human CD4 and CD8 T cells recognize around 22 and 17 HSV-1 proteins, respectively, including enzymes and structural proteins [237,240]. For an excellent review on this see [241]. HSV-specific CD4 T cells express T helper type 1 (Th1)/Th0-like cytokines and have a cytolytic potential [238,242].

One relevant question is how T cells are primed against HSV. Data obtained with animal models indicate that both migratory dendritic cells (DC) present in the skin and DC resident in the draining lymph node present HSV antigens to T cells [243–246]. Langerhans cells (LC), on the contrary, do not prime Th1 cells against HSV [244]. Plasmacytoid DC (pDCs) infiltrate the dermal-epidermal junction within herpes lesions and recruit effector lymphocytes. pDCs are not permissive to HSV productive infection *in vitro* and their exposure to HSV stimulates the proliferation of autologous T cells [247]. Antigen presentation by DC to CD4 T cells results in expression of IFN-γ [246], a relevant cytokine for protection during genital infection in mice as shown with genetic and neutralization studies [248,249]. IFN-γ is involved in clearance of HSV-2 and induction of protection in neurons [250]. It also triggers chemokine expression by epithelial cells in the lesion, leading to recruitment of T and NK cells in animal models [251–253]. The induction of cytokine secretion by CD4 T cells is not due to MHCII-dependent antigen presentation by infected cells, probably because HSV-2 does not replicate efficiently in antigen presenting cells [254] and it reduces MHCII levels [255]. Interestingly, uninfected DC and B cells obtain the antigens from infected epithelial cells and present them to CD4 T cells, enhancing the Th1 response. CD4 tissue resident memory (Trm) cells migrate to the mouse genital mucosa due to the expression of chemokines by macrophages [256] and through the cutaneous lymphocyte antigen (CLA)/E-selectin pair. HSV-2-infected epithelial cells express E-selectin inducing migration and retention in the skin and mucosa of HSV-2 specific T cells that express CLA [257].

T cells are less efficient in controlling colonization of sensory ganglia by HSV-2 than HSV-1 after genital infection of mice [252]. Infection with HSV-1, but not HSV-2, leads to expression of IFN-γ by NK cells 1 day post-infection. This correlated with the earlier appearance of mature DC in the draining lymph node and with a faster activation and dissemination of CD4 and CD8 T cells expressing IFN-γ, required for neuroprotection, in HSV-1 than HSV-2 infected mice [252]. Interestingly, T cell depletion facilitates HSV-1 spread and colonization of neurons while it does not affect HSV-2 infection of sensory ganglia [252]. These results, together with the differences in VP16 promoter indicated above (see “Latency and reactivation”) might help explaining why HSV-2 is more virulent than HSV-1 in these models and in humans during primary genital infection and recurrences (see “Genital herpes”).

HSV-1 establishes latency in sensory neurons of severe combined immune deficiency mice [258], suggesting that the adaptive immune response is not required to drive the virus into latency and that intrinsic and innate immune responses are sufficient. Moreover, HSV-1 establishes latency in mouse DRG despite transplantation of CD8 memory T cells prior to infection. However, the presence T cells reduces the virus replication in the skin and the number of infected DRG neurons, which might limit the extent of reactivation [259], since there is a correlation between virus spread in the ganglia and the number of neurons that become latently infected with the rate of reactivation [260,261]. The ex vivo reactivation rate in mouse TG correlates directly with the viral ganglionic load and inversely with the number of HSV-specific CD8 T cells [262].

The adaptive immune response is required to control latent HSV and to clear the virus upon reactivation. Studies performed with mice indicate that T cells control reactivation from latently infected ganglia. HSV-1-specific CD8 T cells infiltrate the mouse sensory ganglia and their depletion results in higher reactivation in an *ex vivo* explant model [108,109,263]. In humans, HSV-1-specific CD8 and CD4 T cells are also present in infected TG [110,111]. The infiltrating T cells in the human infected TG are oligoclonal, have characteristics of memory effector T cells and surround neuronal cell bodies and axons [110,264]. Memory CD8 T cells express IFN-γ to inhibit HSV replication in neurons and block neuronal apoptosis in mice [265], but they can also release granzyme B granules cleaving ICP4, required for transcription of early genes, instead of triggering apoptosis as they would do in other cell types [266]. This might facilitate the silencing of HSV-1, maintaining latency and neuronal survival [266,267]. It is not clear how CD4 and CD8 T cells recognize the latently infected neurons since viral proteins are not normally expressed during latency and neurons are not efficient antigen presenting cells. One possibility is that, as already mentioned above (“Latency and reactivation”), there is limited viral gene expression allowing recognition by T cells [103]. This, together with a potential low level of neuronal MHC class I expression might allow CD8 T cell recognition and control of reactivation by non-cytolytic mechanisms [263]. Moreover, satellite glial cells in contact with neurons could play such role as antigen presenting cells and control HSV-1 latency without neuronal damage through the expression of T cell inhibitory molecules [268]. The presence of HSV-specific CD8 T cells that could control HSV reactivation in mouse and human sensory ganglia does not match with the frequent reactivation observed in humans [151,269]. Spontaneous reactivation is uncommon in the mouse model of latent infection. The reasons for the discrepancy between the low and high reactivation rates in mice and humans, respectively, are not clear.

T cells are essential to control reactivated HSV in the skin and mucosa. There is a high percentage of HSV-specific T cells in diseased genital skin and in the cornea of patients with recurrent infection [270–272]. HSV-2-specific CD4 T cells have a Trm phenotype and persist in previously infected human skin and genital mucosa and, together with NK cells, express IFN-γ [273]. CD8 T cells infiltrate the human genital mucosa and are linked to HSV-2 clearance from herpetic lesions [270]. CD8 Trm cells locate at the dermal-epidermal junction of the human genital mucosa, in close contact with basal keratinocytes near the nerve endings where HSV-2 exits the neurons after reactivation [234,235,273]. These Trm cells lack expression of chemokine receptors needed for lymphocyte egress and recirculation, have an antiviral expression profile and contain cytolytic granules [235,274]. Trm cells contact several cells with their dendrite-like projections and stay activated in the absence of antigen stimulation [275]. Once a Trm cell recognizes an infected cell it proliferates locally, facilitating control of infection by secreting cytokines, perforin and granzyme B, inducing apoptosis of the target cells [275,276]. The local enrichment of the CD8 Trm cells correlates with episodes of HSV-2 asymptomatic reactivation in humans [235]. These reports indicated that Trm cells located at the dermal-epidermal junction play a key role in immune surveillance and in controlling HSV-2 reactivation in the human genital mucosa [234,235,273,277]. HSV-2-specific effector memory CD4 and CD8 T cells are also enriched in the human cervix of HSV-2-infected women [236,278]. T cell density inversely correlates with symptomatic recurrences [277,279]. Differences in densities and location of T cells and myeloid cells probably influence the different reactivation rates and clinical outcomes observed between patients [280].

Neutralizing antibodies are produced during infection and can block HSV in the neuron-epithelial cell interface [281,282]. *In vitro* experiments with primary human genital epithelial cells showed that Fc receptors facilitate transcytosis of anti-HSV-2 IgG antibodies. *In vivo* experiments with mice supported the notion that IgG transcytosis in the genital tract is important for protection against HSV-2 challenge [283]. Moreover, maternal anti-HSV-2 antibodies reduce neonatal HSV-2 transmission [284]. To achieve antibody-mediated protection in the vaginal mucosa plasma cells must migrate into the tissue since circulating antibodies do not normally enter. This may explain the low success rate of HSV-2 vaccines that elicit a potent antibody response in blood [285]. Unfortunately, plasma cells do not normally colonize the lamina propria of the female mouse reproductive tract following primary HSV-2 infection. However, local vaccination with an attenuated HSV-2 strain followed by challenge with a virulent one results in efficient migration of plasma cells into the genital tract and secretion of HSV-specific antibodies [286]. This occurs due to secretion of IFN-γ by CD4 tissue-resident T cells that trigger the expression of CXCL9, CXCL10 and other chemokines, directing the migration of plasma cells into the vaginal mucosa [286]. Moreover, memory CD4 T cells enter the DRG and spinal cord upon challenge of mice immunized with an attenuated HSV-2 strain lacking expression of the thymidine kinase (TK). Secretion of IFN-γ by the memory CD4 T cells increases the permeability of the blood-brain and blood-nerve barrier, allowing entry of anti-HSV-2 antibodies to the nervous tissue, reducing viral spread [287].

HSV also modulates the adaptive immune response, impacting pathogenesis. HSV productively infects human immature DC *in vitro*, blocks their maturation, impairs their activity, triggers apoptosis and facilitates cross-presentation by non-infected DC [288,289]. HSV inhibits antigen presentation through several mechanisms. For instance, ICP47 blocks antigen presentation [290,291] and ICP34.5 inhibits autophagy, a process involved in antigen presentation [292]. Chemokines are required to recruit leukocytes to the site of infection. HSV gG binds a reduced number of chemokines and enhances their activity *in vitro* and *in vivo*, potentially skewing the type of immune response [293]. The mechanism of action involves modification of chemokine receptor clustering and signaling [13]. HSV infection of activated T cells is not productive, but represses T cell receptor signal transduction and T cell cytolytic activity [294]. HSV can also inhibit the antibody response. For instance, HSV gE and gC bind to the Fc region of antibodies and complement, respectively, inhibiting antibody-dependent cell-mediated cytotoxicity [295]. Moreover, HSV might not be exposed to antibodies during cell-to-cell spread in the mucosa [296]. Interestingly, gC is a type I transmembrane protein that undergoes alternative splicing producing a protein form that lacks the transmembrane region and is secreted, suggesting that it can block complement activity in a paracrine manner [297].

## Herpes stromal keratitis

HSV infection of the eye can cause conjunctivitis, blepharitis, retinitis, epithelial keratitis and herpes stromal keratitis (HSK) [298]. Epithelial keratitis progresses to HSK in about 25% of individuals [299]. The lesions formed during primary infection normally resolve in about one to two weeks due to the action of the immune system [11,300,301]. However, HSV reactivation can result in recurrent infection of the corneal stroma and HSK, characterized by a chronic inflammatory response that can cause neovascularization, corneal scarring, thinning and vision loss [300,301]. For a thorough description of the clinical presentation of ocular disease caused by HSV infection see [11]. HSK is the leading cause of infectious blindness worldwide and the second cause of blindness – after cataract- in the developed world. The global annual incidence of HSV keratitis was estimated to be 1.5 million in 2012, with about 40,000 new cases of blindness or visual impairment occurring every year [302].

The use of animal models, mainly the mouse, has contributed greatly to our understanding of the development of HSK. Reactivation in the mouse is inefficient. Therefore, most results obtained reflect the impact of primary infection in an HSK model, while in humans HSK occurs due to recurrent infection. Despite this difference, the phenotypic similarities observed between the mouse model and disease in humans (i.e. similar type and kinetics of immune response, similar clearance time post-infection) support the validity of many findings obtained with these animal experiments.

HSK is an immune-driven disease [10,11]. The immune response to infection, rather than a cytopathic viral effect, is the major determinant of HSK. It has been suggested that the existence of a pro-inflammatory immune response and an angiogenic milieu is due to HSV latency in the cornea or the presence of HSV DNA or proteins after resolution of an active infection [303]. However, to determine whether this hypothesis is correct requires more investigation. Initial virus replication is also required for HSK as shown with mouse experiments using UV-inactivated virus and replication-deficient HSV [304]. During homeostasis, the number of leukocytes in the cornea is low. Similarly, the healthy cornea is avascular. These two characteristics allow transparency, a requirement for clarity of vision. Increased vessel formation leads to opacity and vision loss. Several angiogenesis and growth factors produced during HSK are responsible for the formation of new blood and lymphatic vessels. Neoangiogenesis, or the production of new blood vessels, is a driving force of the disease since it contributes to cornea opacity and to the recruitment of more leukocytes with inflammatory properties. Angiogenesis can affect the structure of the cornea, increase its opacity and affect light penetration into the retina [301].

The action of the immune system clears primary HSV infection in about 7–14 days. It is not fully understood which element of the immune response is responsible for the resolution of the infection. Detection of HSV by PRR during primary infection leads to expression of IFN, ISG and other cytokines to inhibit virus replication and spread. Type I, II and III IFNs contribute to control HSV in the cornea and facilitate virus clearance [300,305–308] and, together with other cytokines and chemokines, recruit leukocytes to the infected tissue. The main chemokines known to play a role in the mouse model of HSK are CXCL1 and CXCL2 that recruit neutrophils, and CXCL10 that recruits DC, macrophages and T cells. Mice lacking the receptor for CXCL1 and CXCL2 have less infiltration of polymorphonuclear (PMN) cells but no difference in virus replication when compared to the wild type mice, suggesting that PMN cells are not essential for elimination of HSV [309]. On the contrary, experiments that impair CXCL10 function result in higher HSV replication and more disease [310,311]. CD11^+^ DC play also a key role in the recruitment of NK cells and inflammatory monocytes to clear the infection from the cornea [312]. Early upon HSV infection, activated DC and macrophages accumulate in the cornea [313]. NK cells participate in HSV clearance, while depletion of macrophages does not affect virus replication, suggesting that other mechanisms control the infection [312,314]. Depletion of DC leads to higher virus replication, higher leukocyte infiltration and disease [313]. Neutrophils migrate to the infected tissue early during infection and their presence is followed by resolution of infection, suggesting that they play a fundamental role in this process. Indeed, depletion of neutrophils increase viral replication and shedding [315]. However, other reports suggest that neutrophils are not essential for virus clearance [312,316]. The clarification of the role of these leukocytes and other PMN in HSV clearance requires further investigation.

The chronic inflammatory response due to high level of proinflammatory cytokines and leukocyte infiltration can lead to corneal scarring, neovascularization and corneal nerve loss. Many of these cytokines and leukocytes have antiviral activities and help clearing the virus during primary infection. An exacerbated immune response continues after the resolution of infection. The chronicity of the immune response, characterized by high level of pro-inflammatory cytokines and infiltration of PMN cells and T cells is fundamental for HSK development. Blockage of IFN-γ, IL-1, IL-2, IL-17, TNF-α and IL-6 decreases HSK pathogenesis in mice [11]. CD8 T cells clear the virus from the cornea but facilitate neovascularization [317]. CD4 T cells are linked to immunopathology and severity of HSK. Antigen presentation by DC results in recruitment and activation of T cells. Interestingly, the use of a mouse strain with DC deficient in autophagy resulted in lack of activation of CD4 T cells and low disease manifestation, without impacting the innate immune response and with similar level of HSV replication as in the wild type mice [318]. Several results indicate that T cells are the main cells responsible for development of lesions in HSK [10,319]. CD4 T cells enter the cornea normally when virus has been cleared or is present at low titers, and contribute to the chronic phase of disease [300]. CD4 T cells trigger also the second neutrophil infiltration wave [10]. An increase in Th1 and Th17 CD4 T cells and a reduction in regulatory T cells is associated with severe HSK [10]. Th1 CD4 T cells secrete IL-2, IFN-γ and chemokines resulting in higher MHC-II expression and induction of a second wave of neutrophil infiltration that correlates with the onset of symptoms [320–323]. Infiltrating neutrophils secrete free radicals, metalloproteases, cytokines and angiogenic factors that cause tissue damage and corneal opacity [315,324]. IL-17 produced by Th17 cells induces the expression of IL-6, IL-8, matrix metalloproteinases, angiogenic factors and chemokines that recruit PMN cells, increasing corneal inflammation [325–327]. The role of Th17 cells in the progression of HSK has been shown using KO mice lacking the IL-17 receptor and neutralizing antibodies to this cytokine [327]. IL-17 is also expressed in human cornea of HSK patients, supporting its role in this disease [328]. On the contrary, regulatory T cells reduce the exacerbated immune response through several mechanisms, including the expression of IL-10, and control immunopathology during HSK [329,330]. Resident corneal cells also express IL-10, reducing the inflammatory response and disease in HSK [331]. Blockage of IL-10 increases disease, while treatment with this cytokine reduces severity of HSK [331,332].

HSV-1 infection of the mouse cornea and subsequent immune response results in expression of proteins that induce angiogenesis directly or through the expression of angiogenic factors like IL-6, VEGF, fibroblast growth factor 2 (FGF-2), matrix metalloproteinase 9 (MMP-9), hepatocyte growth factor and TNF that trigger lymphangiogenesis and hemangiogenesis [333–335]. The expression of many of these proteins and thereby angiogenesis continues after virus clearance [334,336]. IL-17 increases the expression of VEGF-A, and reduces that of soluble VEGF receptor (sVEGFR), which normally acts as a sink for VEGF-A [325]. Inhibition of VEGF and MMP-9 or reduction of their expression has therapeutic properties in HSK [337–339]. It is not clear whether MMP-9 secreted by infiltrating neutrophils induces angiogenesis leading to HSK. Two independent reports comparing angiogenesis in wild type versus MMP-9 KO mice had opposite results. In one case, angiogenesis was reduced in the MMP-9 KO mice and upon inhibition of MMP-9 in wild-type mice [338], while another study observed no difference [336]. Neutralization of FGF-2 inhibits the expression of other angiogenic factors and maintains clarity of vision [336]. Antibody depletion of neutrophils, monocytes, and a subset of T cells does not reduce angiogenesis, suggesting that infiltrating leukocytes are not the main inducers of neovascularization of the cornea after virus clearance, pointing to a role for resident cells in this process [336]. Therefore, to assign a functional role in HSK neovascularization to specific cytokines, angiogenic factors and cell types requires further investigation. HSV-1 also induces angiogenesis directly, through the action of ICP-4 that triggers the expression of VEGF-A [340].

## Genital herpes

Genital herpes is one of the most prevalent of the sexually transmitted diseases. Most cases of genital herpes occur in people between 16 and 40 years old, correlating with higher number of sexual encounters. HSV-2 is the main virus causing genital herpes, although the number of cases caused by HSV-1 has increased in the last decades, especially in the industrialized world [341,342]. In 2016, about 190 million people aged between 15 and 49 had genital herpes caused by HSV-1, while HSV-2 caused this disease in more than 600 million individuals [1]. In some developing countries HSV-2 prevalence is higher than 50% and this number increases to nearly 100% in risk populations such as commercial sex workers [343]. The disease is more common in women than men, at least in certain geographical locations [344]. Genital herpes can occur during primary infection and reactivation, although primary infections tend to be more severe [345]. However, about 25% of patients that report the first clinical episode were already seropositive for HSV-2, indicating that primary infection was asymptomatic [346,347].

Genital herpes presents with inflammatory lesions in and around the genitals. The anus is also affected, especially in men who have sex with men. The clinical symptoms of genital herpes include fever, muscle pain, itching, headache, myalgia, etc [348]. Initially, papules appear, followed by blisters and lesions that will develop into ulcers. For a more extended description of the clinical presentation of this disease see [347,349]. Infection of the cervix occurs more often during primary infection than upon reactivation, correlating with lower HSV-2 shedding in this anatomical site. Despite this, more than 5% of women participating in a large cross-sectional study had infectious HSV in the cervix [350]. Innate and adaptive immune responses play a role in controlling infection but may also contribute to the establishment of a chronic inflammatory state characteristic of the lesions. Pro-inflammatory cytokines and immune cells are present in these lesions, including T cells, monocytes and macrophages [280,351]. The presence of immune cells susceptible to HIV infection might contribute to HIV acquisition even in the presence of anti HSV therapy [347,352].

HSV-1 recurrences are less frequent and less aggressive than those of HSV-2 [344,353]. This could be due to a lower colonization of the sacral DRG by HSV-1 than HSV-2, despite similar level of replication in the genital mucosa, as suggested by animal studies [354]. Experiments with guinea pigs showed a positive correlation between the number of viral genomes and LAT transcripts and reactivation rates [260,262]. As suggested above (“Adaptive immune response to HSV”), experiments in mice suggest that HSV-1 is less effective than HSV-2 in colonizing the sacral DRG due to faster accumulation of protective T cells at the site of infection and the appearance of mature DC cells at the draining lymph node [252]. Since HSV-1 recurrences are less aggressive than those caused by HSV-2, it is important to determine with a laboratory assay such as polymerase chain reaction (PCR) whether HSV-1 or HSV-2 is the causative agent, in order to tailor the patient counseling [355]. Clinical studies involving daily sampling from the genitalia showed that HSV-2 reactivates very frequently ranging from 20–60% of days in immunocompetent individuals [269,356]. Between 50–80% of HSV reactivations are subclinical and last about 6 hours [150,357]. There is a high variability in the rate and severity of recurrences [280]. As discussed in section “Adaptive immune response to HSV”, the differences in reactivation rate are probably due to different latent viral load and to differences in the innate and adaptive immune responses between individuals.

Genital infection with HSV during pregnancy might result in infection of the fetus or neonate. The risk of transmission to the newborn is higher when infection of a seronegative mother occurs in the third trimester of pregnancy [6,358]. If the mother is seropositive prior to pregnancy, or becomes infected early during pregnancy, the delivery of neutralizing antibodies to the neonate increases protection [359,360]. Recurrent infection poses less risk to the infant [358]. Most cases of infection occur when vaginal secretions containing HSV contact the skin or mucosa of the neonate during delivery [361]. Therefore, cesarean sections prevent HSV transmission during delivery, in particular if the mother is shedding virus [347,358]. Although neonatal herpes is not very common, it is linked to high morbidity and mortality. It can present with local disease in the skin, mucosa, eyes, encephalitis and disseminated disease involving multiple organs [7,347,349]. Encephalitis normally occurs between one and three weeks post-partum and tends to be accompanied by skin blisters [362]. Without treatment, most infants die, in particular those suffering from encephalitis or disseminated disease [359,363]. Survivors of neonatal herpes frequently have sequelae, including peripheral relapses and neurological disease, and these are more severe in infants who had encephalitis caused by HSV-2 than HSV-1 [7,363–365].

The lack of a mature immune system in neonates probably contributes to the severity of HSV infection. Similarly, immunocompromised individuals, including those suffering AIDS, are at higher risk of severe genital herpes. They suffer more severe symptoms of longer duration and have more recurrences than immunocompetent ones [366]. Moreover, the appearance of HSV strains resistant to acyclovir, a guanoside analog that is normally used to inhibit HSV, is higher than in immunocompetent individuals [367].

Epidemiological and clinical studies showed that genital herpes is positively linked to the risk of acquiring and transmitting HIV [368–370]. High HSV-2 seroprevalence is normally accompanied by high number of HIV-infected individuals. For instance, in sub-Saharan Africa, the prevalence of HSV-2 in HIV-infected individuals is very high, between 50% and 90% [371]. Infection with HSV-2 increases the transmission rate and risk of sexually acquiring HIV [372]. The reasons for this are not completely known. One possible explanation points to the presence of HSV-specific CD4 Trm cells that remain in the genital mucosa of individuals with genital herpes for longer periods of time [352,373], to prevent HSV spread following reactivation from sacral ganglia. Many of these CD4 T cells express the chemokine receptor CCR5, which is employed by R5-tropic strains that normally are transmitted between individuals. Moreover, the genital ulcers caused by HSV-2 disrupt the mucosa, facilitating HIV acquisition [371]. Finally, HSV-2 reactivation is linked to higher HIV shedding and thereby transmission [374].

## Herpes simplex encephalitis

HSE is the most common form of sporadic viral encephalitis. With an incidence of HSE between 2 and 4 per million people worldwide, HSV-1 is the virus causing the highest number of viral encephalitis cases in children and adults based on data from several countries, while HSV-2 causes HSE mainly in neonates and immunosuppressed individuals [375–379]. The disease is more common in children and elderly [380]. Approximately 30% of cases occur in children, mainly between 3 months and 6 years of age [3]. Between 20% and 30% of HSE patients die, even after undergoing appropriate treatment [381,382], and mortality increases to 70% in the absence of treatment [383,384]. Children and neonates suffer higher morbidity and mortality rates than adults [379]. Long-term sequelae including behavioral and cognitive disorders affect up to 70% of surviving individuals [383].

The most common symptoms of HSE, occurring in more than 50% of cases are fever, confusion, changes in behavior, headache, impaired mental status, altered consciousness and seizures [385]. For a more detailed description of the clinical presentation of HSE see [362,385]. None of the symptoms are specific of HSE and therefore a fast, efficient and accurate diagnostic test is required. The HSV PCR test amplifying HSV genes from the cerebrospinal fluid (CSF) is the gold standard diagnostic test for HSE [386]. Analysis of the CSF to determine changes in the cell count, intrathecal HSV-specific antibodies, metabolites, etc., and neuroimaging are also employed to diagnose HSE [387]. Some HSE patients suffer relapses after the first episode [388], normally due to HSV replication or to HSE-induced autoimmune encephalitis, a disease that impairs cognitive performance. HSE-induced autoimmune encephalitis occurs after the generation of IgG antibodies to the N-methyl-D-aspartate receptor (NMDAR) or dopamine-2 receptor, through an unknown mechanism [389].

HSE can occur during primary infection or upon reactivation from latency. Some reports suggest establishment of latency and subsequent reactivation in the brain as the cause of encephalitis [362]. However, infection of the brain normally occurs upon neuronal transport of HSV from the periphery. Transmission through the hematogenous route can occur in neonates and immunosuppressed individuals, while other reports indicate that this route is not relevant for HSE [385]. HSV can reach the CNS through the olfactory bulb causing forebrain HSE or through the trigeminal ganglia (TG), causing brainstem HSE [3]. About 95% of reported HSE cases correspond to forebrain HSE [3].

The intrinsic and innate immune responses are key to protect against HSV-1 infection of the CNS and subsequent pathologies, including HSE [162,390–393]. The IFNAR in neurons plays a critical role in protection [197]. The use of drugs that dampen the immune response (e.g. natalizumab) increases the risk of HSE. Interestingly, several clinical and epidemiological observations indicate that HSE is not higher in individuals with cell-intrinsic genetic inborn errors in leukocytes [3,394]. However, CNS intrinsic deficiencies in genes involved in immune control of HSV, such as TLR-3 pathway or the MHC-I allotype, predispose to HSE [395]. These inborn errors tend to display incomplete clinical penetrance and account for approximately 7% of childhood encephalitis [3]. Defects in TLR-3 and type I IFN response in the CNS have been described in approximately 5% of tested children with HSE [396].

In humans, CNS intrinsic inborn errors leading to loss of function of six genes associated with TLR3 pathway – TLR3, UNC93B1, TRIF, TRAF3, TBK1 and IRF3 – predispose to HSE [161,162,390–393,396–398]. Mutations in other TLRs do not seem to predispose to HSE. This correlates with findings that mutations in a protein involved in signaling of all TLRs except TLR3, IL-1 receptor-associated kinase-4, is not linked to HSV infection or HSE [399]. The clinical penetrance of mutations in TLR3 pathway is incomplete. Some relatives of patients suffering HSE that harbor the same mutation in TLR3 do not suffer from disease, indicating that other factors are relevant. These factors could be related to the viral load, route of entry, age, gender or health status of the host. Interestingly, the role of the TLR3 signaling pathway seems specific for HSV-1, since children with these defects are not more susceptible to other viral infections. Moreover, as discussed below, these children develop an efficient immune response against HSV infection in the periphery, indicating that other redundant and TLR3-independent defense mechanisms against HSV are present in the periphery but not in the CNS [390,398]. Indeed, CNS but not PNS neural cells express constitutively active TLR3 [400].

Both non-hematopoietic and hematopoietic resident cells of the CNS play relevant roles in protection. Astrocytes, microglia, oligodendrocytes and neurons express several PRR that detect HSV-1, including TLR3 [180,383,401]. The cGAS-STING pathway in microglia orchestrates the innate response to HSV-1 in a paracrine manner that involves the action of type I IFN and priming of sensing signaling pathways such as TLR3 in other cell types [180]. Lack of STING or cGAS results in lower IFN expression in the brain and higher HSV replication in neurons upon corneal infection of mice, and more HSE [180,402]. Activation of STAT1 in neurons mediated by type I IFN confers resistance to HSV-1 upon corneal infection in mice [197]. The suggested role of microglia in protection, based on mouse experiments, correlates with the presence of activated microglia in the proximity of HSV-1 infected cells in the temporal lobe of HSE patients [403]. Mouse experiments also support a key role for TLR3 pathway in non-hematopoietic cells of the CNS to protect against HSV-1. Both neurons and astrocytes require functional TLR3 to inhibit HSV [404]. These results suggest that TLR3-induced expression of IFN is of particular importance to fight HSV-1 in the human forebrain. Experiments in vitro also showed that mutations in genes within the TLR3-IFN pathway enhance susceptibility to HSV-1 in neurons [396]. This kind of experimental approach also permits investigating mechanistic hypotheses that cannot be addressed with patients. *In vitro* studies with fibroblasts and peripheral blood mononuclear cells from a patient with mutations in IRF3 linked to HSE showed HSV-specific defective signaling in TLR3-TRIF and cGAS-STING pathways [393]. These reports and the fact that HSV-1 targets the cGAS-STING pathway through many mechanisms, supports again the relevance of cGAS-STING to control HSV-1 [175,203,207,209]. The use of iPSC-derived neurons with characteristic of CNS or PNS from patients with inborn errors in the TLR3 pathway suggests that the relevance of this PRR against HSV-1 infection differs between these two types of neurons [400]. Indeed, iPSC-derived cortical neurons and oligodendrocytes with deficiencies in the TLR3 pathway do not control HSV-1 as well as their wild-type counterparts [405]. The iPSC-derived cortical neurons have constitutive TLR3 defense mechanisms against HSV-1, while iPSC-derived sensory neurons do not, they require stimulation of TLR3 or of the IFN pathway to control the virus [400].

Other genetic polymorphisms have been linked to HSE. Patients with X-linked recessive NFκB essential modulator (NEMO) deficiency or autosomal recessive STAT-1 deficiency also suffer syndromic HSE [406,407]. Defects in proteins that are required for production of cytokines including type I and III IFN like STAT1, tyrosine kinase 2 and NEMO are linked to susceptibility to viral infections, including HSE [406,407]. Two adult patients with HSE contained mutations in the central activator of the lectin pathway of the complement system, the mannan-binding lectin serine protease 2 (MASP-2) [408]. MASP-2 binds to PRR like collectins and ficolins that detect mannan and carbohydrate molecules on the surface of HSV and triggers the cleavage of C4 and C2 leading to inflammation and lysis of infected cells. Rare heterozygous variants of the SNORA31 gene, encoding the small nucleolar RNA 31 (SnoRNA31) are associated with forebrain HSE in humans [409]. The predicted function of SnoRNA31 is not directly related to the IFN response, but to direct the isomerization of uridine residues to pseudouridine in ribosomal RNA [410]. Deletions in SNORA31 increased susceptibility of iPSC-derived CNS neurons to HSV-1. Similar results were obtained with cortical neurons derived from iPSC obtained from patients with SNORA31 mutations. Exogenous addition of IFN-β reverted the phenotype. Neurons with mutations in SNORA31 had an impaired response to HSV-1 but not to TLR3 or IFN stimuli, suggesting that the mechanism of action of SnoRNA31 and TLR3 to inhibit HSV-1 differ [409].

There are no major differences in HSE incidence between immunocompetent and immunocompromised individuals but the morbidity and mortality are higher in the latter population [411,412]. Interestingly, children with inborn errors in brain intrinsic immunity develop a potent, specific anti-HSV immune response that protects them from HSV spread to other organs as efficiently as in healthy people [360]. Survivors of HSE due to inborn errors in CNS intrinsic immunity and HSV-1 seropositive individuals who did not suffer HSE have similar CD8 T cell responses to HSV-1 [360]. Therefore, despite having a predisposition to HSE, these children could prime and maintain HSV-1 specific T cell responses as other healthy individuals [360]. These observations support the findings that the genetic disorders causing childhood HSE are CNS-intrinsic and that severe combined immune deficiency (SCID) is not linked to this disease [396,413]. Children with SCID are not at higher risk of HSE than others, but have more severe epithelial infection, suggesting that the adaptive immune response is not the main defense against childhood HSE but is required for protection at the periphery [414]. This is different from neonatal HSE, in which survivors suffer from recurrent HSV reactivation and disease in the periphery and the CNS [365]. Neonates suffering from HSE normally have high viral titers in the mucocutaneous surfaces and in viscera.

Ideally, the immune response should control the virus and then decrease. Otherwise, an uncontrolled and excessive immune response might be detrimental. For instance, damage to the CNS is probably the result of viral replication leading to cytopathic effect and an uncontrolled inflammatory response. Effector CD4 and CD8 T cells infiltrate the human brain to control acute HSV-1 infection of the CNS during HSE [415,416]. This could have detrimental effects for the host since an increase in soluble Fas was observed in the CSF of patients suffering severe HSE, potentially linking the inflammatory response with neuronal cell death and disease outcome [417]. The expression of chemokines and other chemoattractants by activated microglia induces infiltration of peripheral leukocytes. These cells, together with resident cells secrete cytokines to control virus spread in the CNS, and some of them are highly concentrated in the CSF during acute HSE. Markers indicative of T cell activation and intrathecal immune activation increase and remain for long periods of time in many patients [418]. These observations indicate the existence of a chronic inflammatory response that could be pathogenic [418]. Indeed, the inflammatory response can also disrupt the blood-brain barrier (BBB) causing vascular brain edema and hemorrhage. HSV-1 can also directly affect the BBB [419]. Sequelae related to brain inflammation include changes in behavior, lower consciousness and altered cognitive functions.

## Potential link between HSV and Alzheimer’s disease

There is an ongoing discussion on whether infection, in particular with HSV-1, is associated with dementia. Postmortem pathological examination of HSE patients suggests that HSV-1 might have tropism for the limbic system, particularly the temporal lobe and hippocampus [387], which are early sites of damage in a majority of individuals with AD [420]. According to online transcriptome databases, several receptors for HSV-1 are selectively enriched in the hippocampus of adult human brains [421], providing a plausible explanation to why this brain area is more afflicted in HSE patients. Pathologically, surviving HSE patients suffer from memory impairment referred as retrograde and anterograde amnesias, which are linked to neuronal loss in the temporal lobe and basal forebrain, respectively. Hence, it is plausible that HSV-1 initially afflicts the limbic system, then travels to basal forebrain areas, resulting in retrograde and anterograde amnesias [422]. Largely based on these clinical similarities, M J Ball [423,424] proposed that latent HSV-1 reactivates from TG, reaches the hippocampus, basal forebrain and frontal cortex, affecting these brain areas, thereby being one risk factor for AD. This concept was strengthened by Itzhaki and colleagues, who detected HSV-1 DNA in the temporal lobe, hippocampus and frontal cortex from both normal and AD brains, but not in the occipital cortex that is least affected in AD [425,426]. Whether these viral genomes represent latent infection that can reactivate is not known since reactivation was not demonstrated. However, HSV can establish latency and reactivate in human brain organoids [65]. The association between HSV-1 and AD is stronger in individuals carrying the APOE4 allele, one of the strongest genetic risk factors for AD. HSV-1 genome loads in human brains positively correlate with the APOE4 allele frequency [427] and epidemiological studies with human cohorts support a role of HSV-1 in development of AD mainly in individuals carrying this allele [428].

Other studies support the contribution of HSV-1 to AD. For instance, a mutation in PILRA, the HSV-1 gB receptor, that reduces viral entry into macrophages, reduces AD risk in humans [429]. Moreover, studies with cultured neural cells demonstrated that HSV-1 causes amyloid-β (Aβ) fibril formation and deposition and abnormally phosphorylated tau protein [430], hallmarks of AD pathology. Interestingly, HSV-1 viral genomes localized within amyloid plaques in AD brains [431]. Epidemiological studies based on cohorts ranging from Asian to European countries reached the conclusion that antiviral treatment against herpesvirus infections lowers the risk of AD and other types of dementia. Among these studies, a recent report with 530,000 individuals living in Sweden found that people with untreated HSV-1 and VZV infections had 1.5–1.8 times higher risk to develop dementia than the treated controls [432], reinforcing the potential causal link between herpesvirus infection and AD. Currently, two clinical trials evaluating the effects of anti-herpes treatment in cognition decline are ongoing. The outcomes will provide further insights to understand the potential link between herpes virus infection and dementia.

Unfortunately, the HSV-1 infection hypothesis of AD had been largely ignored until recently. The interest on HSV-1 and other pathogens as potential agents contributing to dementia has increased recently, partly due to the lack of success of current therapeutic strategies to treat AD. In 2016 many researchers and clinicians working on AD appealed to the scientific community to investigate the potential link between infectious agents, including HSV-1, and this neurodegenerative disease [433]. Two years later, a systems-level bioinformatic analysis reported that DNA from HSV-1 and human herpesvirus 6 (HHV-6) are specifically enriched in three separate AD cohorts, and discovered that gene networks regulating virus-host interactions are also involved in the AD biology [434]. Another independent study from the same year showed that HSV-1 infection accelerates Aβ deposition in mouse AD brains and in a human 3D neuronal culture model expressing exogenous amyloid precursor protein (APP) [435]. A subsequent study [436] using a 3D human brain-tissue model that was devoid of exogenous factors related to AD, supported the conclusion that HSV-1 infection induces Aβ aggregation, by upregulating the expression of PSEN1 and PSEN2, two core proteins in the γ-secretase complex that generates Aβ. Furthermore, the aggregated Aβ exhibited fibrillar plaque-like formations, reminiscent of the Aβ plaques found in sporadic AD patients. HSV-1 also triggered high levels of neuroinflammation – another risk factor for neurodegeneration – in this model. Since Aβ is part of the innate immune response against pathogens, the current model suggests that it is produced to fight infections in the CNS. Experiments *in vitro* suggest that Aβ binds HSV-1 gB, gC, gD, gE, gH and gG and that Aβ fibrillar structures capture and neutralize HSV-1 and HHV-6 particles [435]. To study Aβ deposition during HSV-1 infection *in vivo*, the authors employed the 5XFAD mouse model that develops Aβ deposition and severe amyloid pathology with age, and infected young mice, when Aβ deposition was still not present [435]. Aβ deposition was detected in the brain of intracranially HSV-1 infected 5XFAD mice, while it was absent from mock-infected and control littermates. A recent report linked infection, inflammation and innate immunity with Aβ deposition. The authors showed that infection and inflammation in the CNS result in high expression of the antiviral interferon-induced transmembrane protein 3 (IFITM3), which binds to and increases the activity of γ-secretase and thereby the production of Aβ [437]. Hence, a possible scenario is that HSV-1 infection not only increases expression of γ-secretase, but also its enzymatic activity through IFITM3, leading to formation of Aβ that acts as an antimicrobial agent and, unfortunately, contributes to AD [435,437].

Many questions still remain. For instance: (1) Infection with HSV-1 is common, but the majority of infected individuals do not develop AD or other neurodegenerative diseases. Does this reflect a possible heterogeneous neurovirulence of different HSV-1 strains that cause differential profiles of neuroinflammation? Or is this due to host factors, particularly genetic risk factors such as the presence of the APOE4 allele, which predispose to dementia upon HSV-1 infection? (2) Is HSV-1 DNA only concentrated around amyloid plaques in Alzheimer’s patients? (3) Which is the major cell type infected by HSV-1 in the brain that plays a role in AD? (4) How does HSV-1 reach certain areas of the brain, particularly the limbic system? The hypotheses are that HSV-1 either directly infects these brain areas through the olfactory bulb during acute infection phase, or after reactivation in the TG. An animal study suggested that the presence of HSV-1 in brain stem correlates with reactivation in TG [93]. (5) Finally, can HSV-1 establish latency and reactivate from human CNS neurons *in vivo*?

## Data Availability

All data has been obtained from peer-reviewed articles that are available at: https://pubmed.ncbi.nlm.nih.gov
